# Next-generation skin wound healing related disease models with integration of immune cells

**DOI:** 10.1093/procel/pwag013

**Published:** 2026-04-08

**Authors:** Yutong Yuan, Shan Zhu, Yuanbo Liu, Yu Yao, Jing Zhang, Xiaoran Li, Yuan Gao, Zilin Zhang, Boyang Song, Jun Ouyang, Juan Zhang, Qiwei Li, Zaozao Chen, Zhonze Gu, Ningbei Yin, Nuo Si

**Affiliations:** Plastic Surgery Hospital, Chinese Academy of Medical Sciences, Peking Union Medical College, Beijing 100144, China; Plastic Surgery Hospital, Chinese Academy of Medical Sciences, Peking Union Medical College, Beijing 100144, China; Plastic Surgery Hospital, Chinese Academy of Medical Sciences, Peking Union Medical College, Beijing 100144, China; Plastic Surgery Hospital, Chinese Academy of Medical Sciences, Peking Union Medical College, Beijing 100144, China; Jiangsu Avatarget Biotechnology Co., Ltd, Suzhou 215163, China; Jiangsu Avatarget Biotechnology Co., Ltd, Suzhou 215163, China; State Key Laboratory of Bioelectronics, School of Biological Science and Medical Engineering, Southeast University, Nanjing 210096, China; Plastic Surgery Hospital, Chinese Academy of Medical Sciences, Peking Union Medical College, Beijing 100144, China; State Key Laboratory of Bioelectronics, School of Biological Science and Medical Engineering, Southeast University, Nanjing 210096, China; Jiangsu Avatarget Biotechnology Co., Ltd, Suzhou 215163, China; State Key Laboratory of Bioelectronics, School of Biological Science and Medical Engineering, Southeast University, Nanjing 210096, China; Jiangsu Avatarget Biotechnology Co., Ltd, Suzhou 215163, China; State Key Laboratory of Bioelectronics, School of Biological Science and Medical Engineering, Southeast University, Nanjing 210096, China; Jiangsu Avatarget Biotechnology Co., Ltd, Suzhou 215163, China; Plastic Surgery Hospital, Chinese Academy of Medical Sciences, Peking Union Medical College, Beijing 100144, China; Jiangsu Avatarget Biotechnology Co., Ltd, Suzhou 215163, China; State Key Laboratory of Bioelectronics, School of Biological Science and Medical Engineering, Southeast University, Nanjing 210096, China; State Key Laboratory of Bioelectronics, School of Biological Science and Medical Engineering, Southeast University, Nanjing 210096, China; Plastic Surgery Hospital, Chinese Academy of Medical Sciences, Peking Union Medical College, Beijing 100144, China; Plastic Surgery Hospital, Chinese Academy of Medical Sciences, Peking Union Medical College, Beijing 100144, China

**Keywords:** organ-on-a-chip, aberrant wound healing, immune microenvironment, pathological scarring, *in vitro* model

## Abstract

Impaired wound healing and pathological scarring remain major clinical challenges, with immune cell dysregulation being a key driver of disease progression. Conventional *in vitro* models fail to recapitulate human immune responses, limiting their translational relevance. In recent years, advances in tissue engineering and microfluidic technologies have driven growing efforts to incorporate immune cells into *in vitro* models, thereby improving their ability to mimic pathological microenvironments. Among these, organ-on-a-chip technology stands out for its capacity to replicate dynamic perfusion, mechanical stimulation, and multicellular crosstalk—features critical for modeling immune-mediated wound repair. This review systematically summarizes recent progress in immune cell-integrated models of aberrant wound healing, including two-dimensional co-cultures, three-dimensional static cultures, organoid systems, and organ-on-a-chip platforms. We highlight core strategies for immune cell integration and their roles in recapitulating key pathological processes such as inflammation and fibrosis. Despite ongoing challenges in cell source stability, model standardization, and long-term culture viability, emerging strategies (e.g., organ-on-a-chip combined with three-dimensional bioprinting or modular design) offer new opportunities for creating biomimetic, high-throughput platforms for wound research. This review aims to facilitate the adoption of immune-integrated *in vitro* models in wound healing research, deepen mechanistic understanding of immune-driven pathology, and accelerate the development of precision therapeutics.

## Introduction

The skin represents not only the largest organ of the human body but also functions as a critical immunological barrier (Quaresma, 2019). Immune cells play indispensable roles throughout the wound repair process when the integrity of this barrier is compromised (Adib et al., 2022). However, disruption of this physiological process can lead to two distinct pathological outcomes: impaired wound healing, as observed in chronic wounds (Wilkinson and Hardman, 2020), or excessive tissue regeneration, clinically manifested as pathological scarring, including keloids and hypertrophic scars (Gauglitz et al., 2011). Both conditions are associated with therapeutic challenges, high recurrence rates, and multifactorial risk profiles. At the mechanistic level, dysregulated immune responses are recognized as key contributors to disease initiation and progression (Berman et al., 2017; Ellis et al., 2018).

In pathological scar tissue, both the abundance and functional activity of immune cells—such as macrophages, T cells, and mast cells—are dysregulated, with concomitant marked elevations in inflammatory mediators, including transforming growth factor beta (TGF-β), interleukin-1 (IL-1), interleukin-6 (IL-6), and tumor necrosis factor alpha (TNF-α) (Shaker et al., 2011; Wang et al., 2020; Yu et al., 2021). In chronic wounds, neutrophils contribute to sustained inflammation through the excessive formation and persistence of neutrophil extracellular traps (NETs). Furthermore, macrophage polarization is impaired, hindering the transition from the pro-inflammatory M1 phenotype to the repair-associated M2 phenotype—a dysfunction that is particularly evident in diabetic wounds. Dysfunction of regulatory T cells (Tregs) also impairs the resolution of inflammation. Meanwhile, the persistent overexpression of pro-inflammatory cytokines such as TNF-α, interleukin-1 beta (IL-1β), and IL-6 further delays wound closure (Mamun et al., 2024). Collectively, this evidence highlights the critical involvement of immune cells in the pathogenesis of aberrant wound repair, underscoring the need for models that can faithfully recapitulate immune-mediated pathological processes.

**Figure 1.**
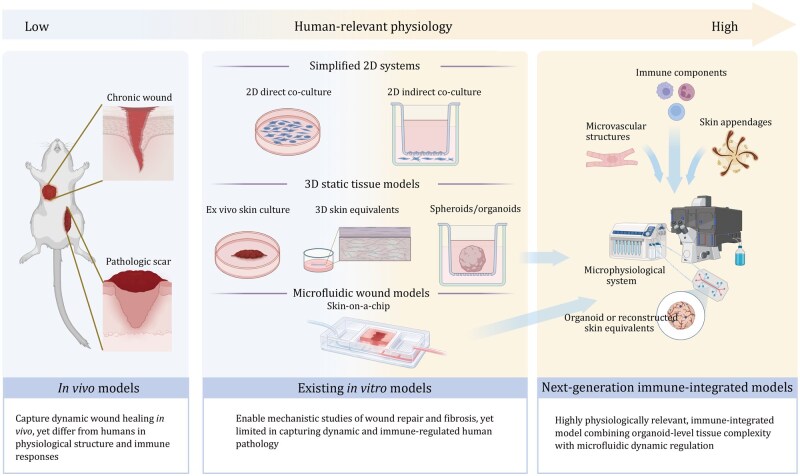
**Currently *in vivo* and *in vitro* skin models for investigating impaired wound healing**. Conventional *in vivo* animal models capture dynamic wound-healing processes but differ substantially from humans in physiological structure and immune responses. Existing *in vitro* skin models enable controlled mechanistic studies of wound repair and fibrosis, yet remain limited in recapitulating the dynamic and immune-regulated nature of human pathological wound healing. In contrast, next-generation immune-integrated platforms combine organoid-level tissue complexity with microfluidic dynamic regulation, offering enhanced physiological relevance and translational potential. Overall, the figure illustrates the progression from simplified models to human-relevant physiology in wound-healing research. Image was created with BioRender.com, with permission. Abbreviations: 2D, two-dimensional; 3D, three-dimensional.

Despite significant advances in experimental wound healing research, current model systems fail to fully recapitulate the complexity of human skin repair and pathological scarring, contributing to persistent translational challenges such as the low success rate of preclinical studies (Ud-Din and Bayat, 2020). While animal models and reductionist *in vitro* models have provided valuable platforms for mechanistic exploration and precision therapies for such diseases (Ud-Din and Bayat, 2017; van den Broek et al., 2014) (Fig. 1), pronounced interspecies differences and simplified cellular compositions limit their translational relevance. In particular, most currently used two-dimensional (2D) and three-dimensional (3D) *in vitro* wound-healing models primarily focus on epithelial and stromal compartments, with limited consideration of immune regulation. The absence of immune components represents a critical limitation from early inflammation to tissue remodeling (Rodrigues et al., 2019). Without immune participation, *in vitro* models cannot adequately recapitulate dynamic inflammatory responses in chronic and non-healing wounds. These constraints contribute to the low predictive value of many preclinical studies (Dai and Chen, 2025).

**Figure 2.**
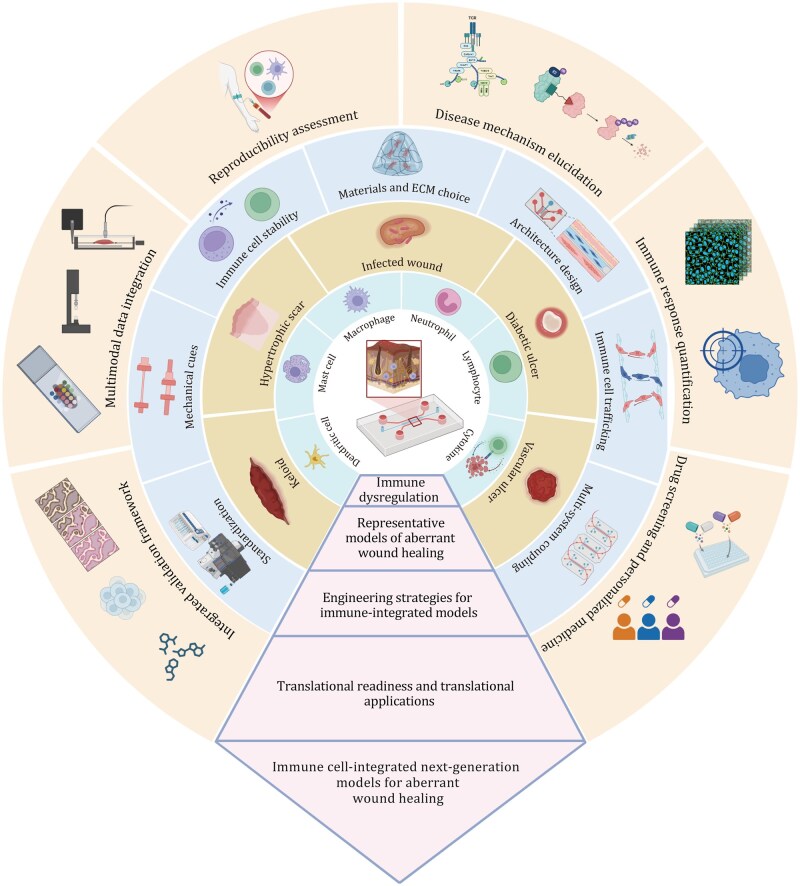
**The conceptual framework of immune cell-integrated models for aberrant wound healing**. This figure depicts a conceptual framework for immune cell-integrated models of aberrant wound healing. From the center outward, the inner layer illustrates immune components that can be incorporated and studied *in vitro*, followed by representative models of impaired wound healing. The next layer outlines engineering considerations for immune-integrated models, while the outer layer highlights translational deployment and applications. Collectively, these interconnected layers define the current landscape and future directions of next-generation immune cell-integrated *in vitro* models for aberrant wound healing. Image was created with BioRender.com, with permission. Abbreviations: ECM, extracellular matrix.

Advanced immunocompetent platforms, including immune-integrated organoid models (Mukhopadhyay, 2024) and organ-on-a-chip systems (Morrison et al., 2024), offer unique opportunities to recapitulate dynamic immune interactions, capture interindividual variability, and improve translational relevance, thereby holding strong promise for enabling more physiologically faithful modeling of wound-healing pathology. However, there remains a lack of comprehensive reviews that summarize the application of immune cells in *in-vitro* models of aberrant wound healing. This article aims to provide an overview of the strategies employed to integrate immune cells into wound healing models, to critically evaluate the strengths and limitations of existing approaches (Table 1), and to outline potential directions for future research. Particular attention is given to engineering considerations, including biomaterial selection, structural design, and biological characterization, which critically influence model fidelity and reproducibility. By doing so, this review aims to provide innovative theoretical perspectives that can facilitate the development of advanced next-generation models (Fig. 2) specifically designed for in-depth mechanistic investigations within this field.

**Table 1.** Summary of representative *in vitro* and *in vivo* immune cell-integrated models for investigating aberrant wound healing.

Model type	Pathological/physiology process simulated	Model construction method	Immune cell manipulation strategy	Immune cell type	Scientific question addressed	Limitations	Reference(s)
***In vitro* models**							
**2D co-culture models**	Wound healing	Transwell co-culture of HDFs and THP-1-derived macrophages	PMA-induced M0; IFN-γ + LPS-induced M1; IL-4 + IL-13-induced M2; flow cytometry enrichment	Macrophages	Paracrine effects of M1/M2 macrophages on fibroblast fibrosis	Differences between THP-1 and patient-derived macrophages; sorting-induced cell stress; short culture duration	Zhu et al. (2017)
	Wound healing	Transwell co-culture of HDFs and CD206⁺ macrophages	PBMC-derived macrophages, M-CSF-differentiated	Macrophages	Regulation of fibroblast fibrosis by M2 macrophages via IL-6	Static culture; non-physiological immune cell ratios	Kurachi et al. (2021)
	Hypertrophic scar	Direct co-culture of HDFs and RMC-1 mast cells	RMC-1 mast cells treated with degranulation inhibitor/gap junction inhibitor	Mast cells	Role of mast cell-fibroblast gap junctions in fibrosis	Rat-derived immune cell line; no mechanical stimulation	Foley and Ehrlich (2013)
	Hypertrophic scar	Transwell co-culture of HDFs and THP-1-derived M2 macrophages	PMA-induced M0; IL-4 + IL-13-induced M2	Macrophages	Regulation of fibroblast activity by M2 exosomal lncRNAs	Non-disease-derived HDFs; short culture duration	Zhu et al. (2021)
	Hypertrophic scar	Transwell co-culture/conditioned medium treatment of scar-derived/normal HFBs with c-Maf-transfected RAW264.7 macrophages	c-Maf plasmid transfection	Macrophages	c-Maf-induced M2 polarization regulates fibrosis via TGF-β1	Mice cell line; short culture duration; unidirectional signaling	Yang et al. (2024)
	Hypertrophic scar	Direct co-culture of scar-derived HFBs and activated Th cells	Murine splenic/lymph node CD4⁺ T cells; polarized into Th1/Th2	Th cells	Regulation of fibrosis by circRNA-mediated Th1/Th2 balance	Cross-species co-culture; not fully consistent with human polarization	Qi et al. (2024)
	Keloid	Direct/Transwell co-culture of KFs and HMC-1 mast cells	HMC-1 cell line	Mast cells	Hypoxia-driven mast cell-fibroblast contact regulates HIF-1α/VEGF signaling	Tumor-derived cell line; acute hypoxia not fully recapitulating chronic disease processes	Zhang et al. (2006)
	Keloid	Transwell co-culture of HaCaT and PBMCs	PBMCs from keloid patients	PBMCs (mainly lymphocytes)	IL-33/IFN-γ feedback loop regulates epidermal inflammation	Immortalized keratinocytes; static culture; heterogeneous PBMC population	Chen et al. (2025)
	Keloid	Direct/Transwell co-culture of KFs and CD8⁺ T cells	Patient PBMC-derived CD8⁺ T cells; CD3/CD28 bead-activated	T cells	Effects of CD8⁺ T cells on fibroblast apoptosis and activation	Static culture; absence of mechanical stimulation	Shan et al. (2022)
	Keloid	Direct co-culture of fibroblasts and Treg cells	Patient PBMC-derived Treg cells; CD3/CD28-activated	Treg cells	Treg cells regulate collagen synthesis via TGF-β	PBMC adherence-derived fibroblast-like cells (stromal cell contamination); short-term culture	Chen et al. (2019)
	Keloid	KF co-culture with M1/M2 macrophage-conditioned media	MACS-enriched CD14⁺ monocytes; GM-CSF + IFN-γ + LPS-induced M1; GM-CSF + IL-4 + IL-13-induced M2	Macrophages	Differential regulation of KF fibrosis by M1/M2 macrophages	Non-disease-derived macrophages; lack of cell–cell contact	Dirand et al. (2024)
**3D static models**	Wound healing	3D collagen gel model with embedded M2 macrophages/fibroblasts	PMA-induced M0; IL-4 + IL-13-induced M2a; IL-10-induced M2c	Macrophages	M2 macrophage regulation of repair initiation/resolution	Static culture; limited ECM components; tumor-derived cell line	Sapudom et al. (2021)
	Wound healing	Scaffold-free 3D human dermal equivalent (endogenous ECM); full-thickness circular wounds (punch biopsy)	PMA-induced M0; IL-4+IL-13-induced M2a; M2a deposited on/infiltrated into wounds	Macrophages	M2a role in fibroblast activation, ECM remodeling, collagen alignment in scar formation	Static culture; no epidermal/vascular compartments; tumor-derived cell line	Passariello et al. (2025)
	Diabetic wound	Transwell-based skin equivalent (type I collagen gel) with patient/healthy fibroblasts/monocytes; keratinocytes seeded on top	MACS-enriched CD14⁺ monocytes; IFN-γ + LPS-induced M1; IL-4 + IL-13-induced M2a; IL-10-induced M2c	Macrophages	Diabetes-specific immune-matrix interactions in chronic inflammation	Mixed macrophage polarization phenotypes; limited sample size	Smith et al. (2021)
	Keloid	MatriDerm^®^ scaffold-based skin equivalent with patient/healthy fibroblasts/monocytes; keratinocytes seeded on top	MACS-enriched CD14⁺ monocytes	Monocytes	Monocyte role in keloid formation	Restricted direct cell contact	Limandjaja et al. (2019)
**Spheroids**	Hypertrophic scar	3D spheroid model (agarose hydrogel) with assembled fibroblasts/macrophages	PBMC-derived macrophages (M1/M2 mixed)	Macrophages	Development of physiologically relevant fibrosis models for drug screening	Undefined macrophage phenotypes; no vascularization/mechanical stress; short culture duration	Tan et al. (2020)
**Organ-on-chip models**	Wound healing	Microfluidic 3-channel chip: HUVECs (Matrigel, central); fibroblasts/macrophages (adjacent)	Healthy donor PBMCs; M-CSF-induced M0; LPS + IFN-γ-induced M1; IL-4 + M-CSF-induced M2	Macrophages	Multicellular interactions/macrophage polarization/angiogenesis in early wound healing	No keratinocytes; short culture duration	Biglari et al. (2019)
***In vivo* models**							
**Animal models**	Diabetic wound	STZ-induced diabetic mice; excisional wound model (punch biopsy)	Mast cell-deficient mice (WBB6F1/J-KitW/KitW-v)	Mast cells	Mast cell role in diabetic wound healing; degranulation inhibitor reversibility	Off-target phenotypes; contradictory results; compensatory mechanisms	Tellechea et al. (2016)
	Diabetic wound	Db/db mice; excisional wounds (punch biopsy)	Local injection of PBMC-derived M2a (IL-4)/M2c (IL-10) (days 1,3)	Macrophages	Exogenous M2 macrophage effects on wound healing	Species-specific leptin signaling; incomplete temporal coverage; wound contraction differs from human re-epithelialization	Jetten et al. (2014)
	Diabetic wound	Db/db mice; excisional wounds (punch biopsy)	Intraperitoneal anti-neutrophil serum injection	Neutrophils	Neutrophil role in wound healing/re-epithelialization	Non-specific immune depletion	Dovi et al. (2003)
	Delayed wound healing	Corticosteroid-resistant guinea pigs; linear dorsal incisions	Hydrocortisone + anti-macrophage serum	Macrophages	Macrophage role in wound repair	Incomplete macrophage depletion; corticosteroid-associated side effects; unclear causal attribution	Leibovich and Ross (1975)
	Delayed wound healing	Mice; excisional wounds (punch biopsy)	CD11b-DTR mice + diphtheria toxin	Macrophages	Macrophage depletion effects on healing	Broad CD11b expression across myeloid cells; acute immune cell depletion model not fully recapitulating chronic disease processes	Mirza et al. (2009)
	Delayed wound healing	Mice; excisional wounds (punch biopsy)	CD11c-DTR mice + diphtheria toxin	Dendritic cells	CD11c⁺ DC role in wound repair	CD11c⁺ population heterogeneity; diphtheria toxin side effects; microbiota variations	Li et al. (2021)
	Delayed wound healing	Mice; excisional wounds (punch biopsy)	Foxp3-DTR mice + diphtheria toxin	Treg cells	Treg cell regulation of wound repair via EGFR pathway	Diphtheria toxin side effects; species-specific differences	Nosbaum et al. (2016)
	Hypertrophic scar	Immunocompetent mice; burn injury + ear skin grafts	None (intact immune system)	Macrophages, mast cells	Development of HS model in immunocompetent hosts	Species-specific skin structure differs; accelerated graft contraction	Ibrahim et al. (2014)
**Humanized animal models**	Hypertrophic scar	Nude mice; human split-thickness skin grafts	Intraperitoneal clodronate liposomes injection	Macrophages	Macrophage role in human HS formation	Non-specific macrophage depletion; murine macrophage infiltration; technically demanding	Zhu et al. (2016)
	Keloid	NSG mice; keloid tissue grafts	Patient/healthy donor PBMCs Injection	PBMCs (CD4⁺ T cells)	Development of humanized keloid model with patient immune cells	Incomplete immune reconstitution; species-specific microenvironment; limited long-term stability	Lee et al. (2023)

Abbreviations: HDF, human dermal fibroblast; HFB, human fibroblast; KF, keloid fibroblast; PBMC, peripheral blood mononuclear cell; THP-1, human monocytic leukemia cell line THP-1; RAW264.7, murine macrophage cell line RAW264.7; RMC-1, rat mast cell line-1; HMC-1, human mast cell line-1; HaCaT, immortalized human keratinocyte cell line; HUVEC, human umbilical vein endothelial cell; ECM, extracellular matrix; M-CSF, macrophage colony-stimulating factor; GM-CSF, granulocyte-macrophage colony-stimulating factor; PMA, phorbol 12-myristate 13-acetate; LPS, lipopolysaccharide; IFN-γ, interferon-gamma; IL, interleukin; TGF-β, transforming growth factor-beta; HIF-1α, hypoxia-inducible factor-1 alpha; VEGF, vascular endothelial growth factor; Th, T helper cell; Treg, regulatory T cell; STZ, streptozotocin; EGFR, epidermal growth factor receptor; MACS, magnetic-activated cell sorting; NSG, NOD scid gamma; DTR, diphtheria toxin receptor.

## The role of immune cells in aberrant wound repair

### Composition and functions of skin immune cells

As a critical immunological barrier, the skin contains a diverse and highly specialized array of immune cells, which are distributed across distinct anatomical layers and perform distinct functions during wound repair.

Within the epidermis, Langerhans cells function as the primary antigen-presenting cells. They can recognize and process exogenous antigens, thereby initiating adaptive immune responses while maintaining immune tolerance. Furthermore, through direct interactions with keratinocytes, Langerhans cells support epithelial repair (Deckers et al., 2018). Moreover, epidermal T cells contribute to wound healing by secreting keratinocyte growth factors and other cytokines, which enhance keratinocyte proliferation (Jameson et al., 2002).

The dermis, as the layer with the most abundant immune cell infiltration, contains macrophages, dendritic cells (DCs), mast cells, lymphocytes, and neutrophils, among others. Neutrophils serve as the first line of cellular defense during the early phase of wound healing. Upon recruitment to the injury site, they eliminate pathogens through the release of reactive oxygen species and cytotoxic granules, as well as through the formation of NETs. Importantly, neutrophils also express vascular endothelial growth factor (VEGF), thereby accelerating wound closure (Kovtun et al., 2018). DCs capture antigens and migrate to regional lymph nodes to initiate adaptive immune responses. During tissue repair, they secrete proinflammatory cytokines and chemokines that promote cell proliferation, granulation tissue formation, and neovascularization (Vinish et al., 2016). Macrophages play an essential role throughout all stages of tissue repair. They not only remove necrotic cells and pathogens but also secrete a range of growth factors, such as VEGF and TGF-β. In addition to their reparative functions, macrophages critically regulate both the initiation and resolution of inflammation. The phenotypic subsets, abundance, and interactions of macrophages with other immune cells largely determine the outcome of wound repair (Krzyszczyk et al., 2018). Mast cells, upon activation, release histamine, prostaglandin D2, and other mediators that coordinate inflammatory cell recruitment and modulate local immune responses. They also promote fibroblast-mediated collagen synthesis, which, while beneficial for tissue repair, may concurrently contribute to excessive scar formation (Wilgus and Wulff, 2014). T lymphocytes are widely distributed throughout the dermis, where they participate in immune surveillance and defense. Distinct subsets perform diverse roles in tissue repair; for example, invariant natural killer T (iNKT) cells enhance wound closure by modulating the temporal dynamics of neutrophil infiltration (Tanno et al., 2017), whereas Tregs inhibit excessive TGF-β production and collagen deposition through interleukin-10 (IL-10) secretion, thereby reducing the risk of pathological scarring (Haertel et al., 2018). Additionally, populations of B lymphocytes, eosinophils, and basophils reside in the skin, contributing to immune surveillance, anti-infective defense, and the maintenance of immune homeostasis. Collectively, this complex network of immune cells ensures that wound healing progresses in a coordinated and efficient manner.

### Immunological characteristics of impaired wound healing

During the process of normal wound healing, immune cell activity must be precisely regulated in both spatial and temporal dimensions. Disruption of this regulatory balance can lead to delayed tissue repair, or the formation of pathological scars.

In chronic wounds, neutrophils fail to undergo apoptosis appropriately and persist at the injury site, sustaining inflammation through excessive NET formation and protease release (Wong et al., 2015). Macrophage abundance and the balance of their polarization states are critical determinants of healing outcomes. Prolonged macrophage accumulation and a defective transition from the pro-inflammatory M1 phenotype to the reparative M2 phenotype leads to sustained overexpression of inflammatory mediators—including IL-1, TNF-α, and inducible nitric oxide synthase (iNOS)—thereby maintaining tissues in a persistent inflammatory state and obstructing normal progression into the proliferative and remodeling phases. Conversely, excessive M1 to M2 polarization promotes excessive extracellular matrix (ECM) deposition and the formation of pathological scars (Hassanshahi et al., 2022; Hesketh et al., 2017). The role of mast cells in wound healing is multifaceted. Mast cell depletion during the early stages of tissue repair reduces vascular permeability and the recruitment of inflammatory cells, thereby impairing the healing process. However, excessive activation of mast cells in chronic wounds also hinders effective repair (Dong et al., 2020), Moreover, excessive mast cell activation and degranulation may contribute to the development of pathological scars through the release of pro-inflammatory mediators such as TNF-α and IL-1 (Dong et al., 2014). Dysregulation of T cell subsets represents another hallmark of chronic wounds and pathological scarring. In chronic wounds, CD4^+^ T helper (Th)1, Th17, and Th22 cells are significantly increased, whereas Tregs are markedly decreased (Boothby et al., 2020). Elevated levels of CD4^+^ Th2 and Th17 cells, along with an increased CD4^+^/CD8^+^ T cell ratio, are strongly correlated with the formation of pathological scars (Wu et al., 2020). Furthermore, reductions in Langerhans cells, DCs, and eosinophils have been observed in diabetic chronic wounds, which may impair local immune regulation and hinder tissue repair (Joshi et al., 2020).

**Figure 3.**
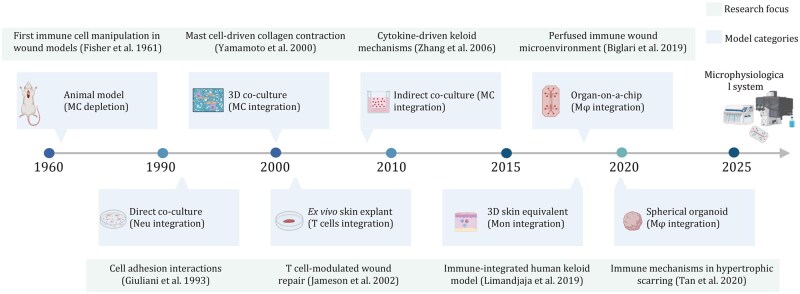
**Timeline of studies incorporating immune cells into various wound healing models**. This figure summarizes representative first-in-class studies introducing immune cells into major wound healing model platforms, spanning early reductionist systems to organoid and organ-on-a-chip approaches. The figure also notes the experimental questions addressed in these reports, capturing diverse facets of immune-tissue crosstalk relevant to repair and fibrotic skin pathology. Image was created with BioRender.com, with permission (Biglari et al., 2019; Fisher and Hellstrom, 1961; Giuliani et al., 1993; Jameson et al., 2002; Limandjaja et al., 2019; Tan et al., 2020; Yamamoto et al., 2000; Zhang et al., 2006). Abbreviations: 3D, three-dimensional; MC, mast cell; Neu, neutrophil; Mon, monocyte; Mφ, macrophage.

Taken together, these pathological immune signatures have progressively driven the incorporation of immune components into experimental wound models. The field has evolved from *in vivo* animal models to *in vitro* cell culture systems and, more recently, organ-on-a-chip platforms (Fig. 3). This evolution mirrors expanding research objectives aimed at capturing increasingly complex aspects of immune regulation in aberrant wound healing.

## Existing models of aberrant wound healing that integrate the immune microenvironment

### Animal models

Chronic wounds—such as diabetic ulcers, pressure ulcers, and arterial or venous leg ulcers—are characterized by complex, multifactorial pathophysiological mechanisms, and related research has traditionally relied on animal models. Commonly used models include the rabbit ear ulcer model, flap ischemia model, ischemia-reperfusion model, and diabetes-induced injury model. These are typically established through full-thickness skin excisions combined with ischemic, mechanical, or metabolic interventions to mimic disease conditions (Nunan et al., 2014). In hypertrophic scar research, animal models are generated by excising full-thickness skin along with the underlying periosteum or by applying mechanical tension to delay wound contraction (Huang et al., 2021; Son and Hinz, 2021). In contrast, animal models for keloids remain inadequate, as their clinical features show limited correlation with those observed in humans. As a result, scar tissue induced in animals through incisional wounds and environmental manipulation more closely resembles hypertrophic scars than keloids (Khorshid, 2005; Seo et al., 2013).

To investigate the role of immune cells through animal models, researchers frequently utilize genetic strategies, such as knocking out essential genes involved in immune cell development and survival or inserting toxin-encoding genes downstream of cell-specific promoters, which result in selective depletion or functional inhibition of the targeted immune cell populations (Fisher and Hellstrom, 1961; Mirza et al., 2009; Nosbaum et al., 2016; Tellechea et al., 2016). Alternatively, specific immune cell subsets can be depleted through the administration of antibodies or pharmacological agents (Dovi et al., 2003; Leibovich and Ross, 1975). Although these approaches are valuable for elucidating immune cell functions, they are constrained by off-target effects, compensatory responses, and a lack of precise temporal control. For instance, acute depletion models fail to adequately mimic chronic dysfunction, and conflicting findings are occasionally reported across studies (Jetten et al., 2014).

To reduce species-related discrepancies, humanized animal models have been developed. These models involve transplanting human keloid-derived cells (Zhang et al., 2009) or tissues (Waki et al., 1991), hypertrophic scar tissues (Li et al., 2021), or split-thickness skin grafts (Zhu et al., 2016) onto animal hosts, often in combination with external pressure or specific factors to maintain pathological scar phenotypes. Such models partially address interspecies differences and offer a more accurate representation of human pathological scarring. However, the local microenvironment in animal hosts differs from those in humans, and the fidelity of these models declines over time, limiting their ability to fully elucidate scar pathogenesis. Moreover, to avoid immune rejection, these models typically rely on immunodeficient mice, whose immune microenvironment diverges substantially from that of humans, thereby affecting the translational relevance of findings (Ibrahim et al., 2014; Lee et al., 2023; Zhu et al., 2016).

In summary, significant differences persist between animal and human skin in terms of structural organization, wound repair mechanisms, and immune responses. Currently, no single animal model can fully replicate human aberrant wound healing. Therefore, model selection should be guided by the specific research objective, and animal systems are most effective when combined with *in vitro* approaches to improve translational relevance.

### *Ex vivo* skin tissue culture

*Ex vivo* skin tissue culture entails the preparation of fresh wound or scar tissue into explants measuring 3–6 mm in diameter, embedding these explants in type I collagen gels or serum-free media, and maintaining them at the air-liquid interface to simulate the native skin microenvironment. The duration of culture can extend up to six weeks. *Ex vivo* cultures derived from hypertrophic scars (Li et al., 2016), keloids (Bagabir et al., 2012), and healing skin (Kratz, 1998) preserve diverse resident cell populations, ECM components, and three-dimensional architecture, thereby serving as valuable platforms for investigating mechanisms underlying wound repair and scar formation. By adjusting explant dimensions, culture medium formulation, and the structural of the culture system, long-term *in vitro* maintenance can be effectively tailored to meet specific experimental objectives.

However, this model has several limitations. First, the absence of a blood circulation system necessitates nutrient and metabolite exchange through diffusion. Second, inter-individual variability among tissue donors may compromise the reproducibility of experimental outcomes. Critically, immune cells are difficult to sustain over extended periods in *ex vivo* cultures. During cultivation, immune cells often migrate out of the tissue or undergo apoptosis, resulting in reductions in both cell numbers and functional phenotypes. A representative example is provided by Duong et al. (2005), who embedded adult keloid tissue explants in a collagen-rich gel matrix for up to six weeks. Their results showed a marked decline in mast cell numbers and an almost complete loss of detectable Langerhans cells, underscoring the intrinsic challenges of maintaining the immune microenvironment without *in vivo* circulatory support.

### 2D co-culture systems

2D co-culture models represent one of the most widely used *in vitro* models for studying skin pathological processes. Based on the mode of cell contact, 2D co-culture systems can be broadly categorized into two types. The first type is the direct co-culture system, in which distinct cell types are seeded together within the same culture plate, allowing for direct physical contact (Giuliani et al., 1993; Qi et al., 2024). For example, Foley and Ehrlich (2013) co-cultured human dermal fibroblasts with the rat mast cell line RMC-1 and demonstrated that mast cells enhanced fibroblast activity through gap junction-mediated signaling, thereby promoting the progression of hypertrophic scar formation. This illustrates the ability of direct co-culture systems to uncover contact-dependent mechanisms of the diseases. The second type is the indirect co-culture system, most commonly implemented using the transwell assay. Different cell populations are physically separated by a semipermeable membrane, which permits the diffusion of soluble mediators—such as cytokines and chemokines—while preventing direct physical interaction, thus enabling the analysis of paracrine signaling pathways. Using this method, Kurachi et al. (2021) examined the antifibrotic effects of CD206^+^ macrophages in pathological scarring by placing macrophages in the upper transwell insert and fibroblasts in the lower chamber. Their findings revealed that CD206^+^ macrophages modulate the IL-6 signaling pathway in a paracrine manner, which subsequently reduced fibroblast-mediated fibrogenic activity.

In pathological scar formation and wound healing research, 2D co-culture systems are widely used to investigate the crosstalk between immune cells and skin-resident cells such as fibroblasts and keratinocytes. Studies have shown that macrophage subtypes can regulate fibroblast fibrotic activity through paracrine signaling (Zhu et al., 2017; Zhu et al., 2021), while keratinocyte and peripheral blood mononuclear cell co-culture models have revealed cytokine-driven feedback loops that contribute to the persistence of chronic inflammation in keloids (Chen et al., 2025). In addition, 2D co-culture systems can be employed to evaluate how resident cutaneous cells influence immune cell function (Chen et al., 2019). These studies provide critical insights into the mechanisms through which immune cells contribute to scar formation and wound healing. Nevertheless, the 2D co-culture model has inherent limitations. Due to the lack of spatial architecture and 3D ECM support, it is unable to accurately recapitulate cell-matrix interactions or cellular responses to mechanical tension, cytokine gradients, and other complex environmental cues.

**Figure 4.**
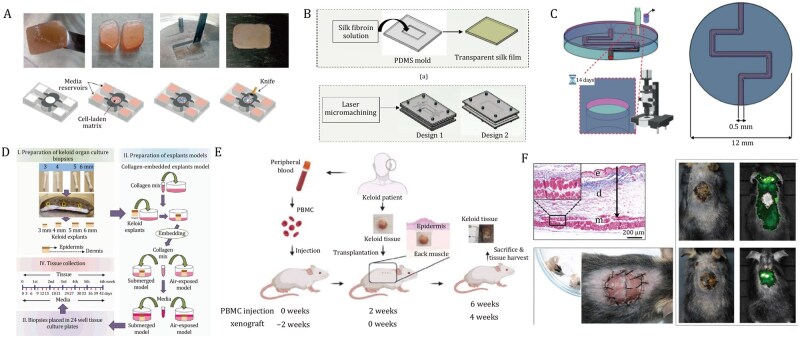
**Construction approaches for aberrant wound-healing models**. (A) A three-dimensional wound-healing model constructed by cutting and repositioning skin equivalents to mimic wound closure. Source: Reproduced with permission from ref. (Urciuolo et al., 2022) Copyright 2022, MDPI. (B) A chip-based wound-healing model established through trypsin injection and the use of a prefabricated polydimethylsiloxane barrier. Source: Reproduced with permission from ref. Gupta et al. (2022) Copyright 2022, MDPI. (C) A chip model in which wounds are simulated by punching holes to observe subsequent tissue repair. Reproduced with permission. Source: Reproduced with permission from ref. Maggiotto et al. (2025) Copyright 2025, Elsevier. (D) A keloid explant model generated by culturing excised keloid tissue *in vitro*. Source: Reproduced with permission from ref. Bagabir et al. (2012) Copyright 2012, John Wiley and Sons. (E) A humanized keloid animal model. Source: Reproduced with permission from ref. Lee et al. (2023) Copyright 2023, Springer Nature. (F) An *in vivo* hypertrophic scar model established by transplanting human split-thickness skin onto full-thickness dorsal skin defects. Source: Reproduced with permission from ref. Ibrahim et al. (2014) Copyright 2014, John Wiley and Sons.

In wound healing research, 2D models are most commonly used to assess keratinocyte or fibroblast proliferation and migration. A widely utilized method is the scratch assay, in which a “wound” area is created within a confluent cell monolayer using a pipette tip, followed by monitoring of the migration and closure dynamics to evaluate the effects of specific interventions (Brem et al., 2008; Wolf et al., 2009). However, this assay differs significantly from the physiological wound healing process, as it only captures basic migratory and proliferative behaviors and fails to replicate immune cell infiltration, angiogenesis, or complex multicellular interactions (Fig. 4).

Taken together, the 2D co-culture system serves as a classical *in vitro* model for investigating the role of immune cells in aberrant wound healing. However, to improve clinical relevance and enhance translational potential, findings obtained from 2D systems should be complemented with data from 3D culture models and tissue-engineered models.

### 3D static culture models

With advances in ECM composition and scaffold technologies, 3D full-thickness skin models that incorporate key cellular components have been developed, and immune cells are increasingly integrated into these systems to more effectively investigate the mechanisms underlying chronic wound healing and pathological fibrosis (Pupovac et al., 2018; Yamamoto et al., 2000). For example, Lim et al. (2024) embedded fibroblasts and macrophages within matrix gels to simulate their dermal distribution, revealing fibroblast-induced M1-to-M2 macrophage polarization and the profibrotic effects of M2 macrophages. However, macrophage viability decreased by approximately 50% after 8 days of culture, highlighting the limitations of static culture systems in sustaining immune cell function. Similarly, Kühbacher et al. (2017) developed a fungal infection chronic wound model by constructing a full-thickness skin equivalent on transwell inserts, with an underlying CD4^+^ T cell-hydrogel layer that enabled cytokine-mediated communication with the skin tissue. Although not specifically designed for wound healing studies, this model demonstrated the potential of spatial compartmentalization in recapitulating immune-skin interactions. Nevertheless, the absence of T cell subset isolation and functional analysis constrained the interpretability of their findings.

In studies of chronic wounds and pathological scars, immune cells are commonly integrated into 3D skin models through either direct embedding or physically separated configurations. The former approach is exemplified by embedding macrophages with fibroblasts in a collagen matrix to investigate their role in ECM remodeling (Sapudom et al., 2021). Similarly, monocytes derived from diabetic patients have been co-cultured with fibroblasts and subsequently covered with keratinocytes to form a stratified epidermis, facilitating the development of models that more accurately recapitulate the microenvironment associated with diabetic foot ulcers (DFUs) (Smith et al., 2021). Some studies avoid direct premixing of macrophages and fibroblasts, instead introducing macrophages after injury and allowing their gradual infiltration into a pre-established, fibroblast-derived dermal equivalent. This strategy minimizes immune cell interference during dermal equivalent construction and more faithfully reflects the temporal sequence of post-injury immune recruitment during wound healing (Passariello et al., 2025). The alternative approach involves spatial segregation through transwell systems. For example, a full-thickness keloid skin model can be reconstructed in the upper chamber, while polarized macrophages are seeded in the lower compartment to simulate the paracrine regulation of keloid by immune cell-derived factors (Limandjaja et al., 2019). In addition, acellular 3D scaffolds have been developed to specifically investigate immune cell functions. Zhou et al. (2022) seeded macrophages onto scaffolds composed of type I collagen and hyaluronic acid to examine how mechanical stretching modulates macrophage function. Moreover, by incorporating T cells into the same scaffold, they demonstrated that direct macrophage-T cell interactions under biomechanical stress critically regulate T cell functionality.

Although 3D static models provide valuable insights into immune cell-matrix interactions, the maintenance of chronic inflammation, and aberrant repair mechanisms, they also exhibit significant limitations. Static culture conditions lack hemodynamic forces and continuous immune cell infiltration, thus failing to replicate dynamic *in vivo* recruitment processes. A study demonstrated that monocyte-derived macrophages exposed to fluid shear stress exhibit distinct morphologies and cytokine secretion profiles compared to those in static cultures, highlighting the significance of biomechanical factors in pathological wound healing (Jui et al., 2024). Furthermore, most models include only one or two immune cell types, overlooking other key contributors such as mast cells and DCs, which play critical roles in chronic inflammation and fibrosis. A study has aimed to introduce linear incisions into 3D skin models and reposition the two incised portions in close apposition to simulate surgical wound healing, with the closure process subsequently monitored in real time. (Lombardi et al., 2017). However, this approach primarily reflects linear incisional repair and cannot be generalized to chronic wounds. Moreover, flap apposition fails to replicate the contractile dynamics of *in vivo* wound closure, limiting the broader applicability of such models (Urciuolo et al., 2022).

### Spheroids and organoid models

**Figure 5.**
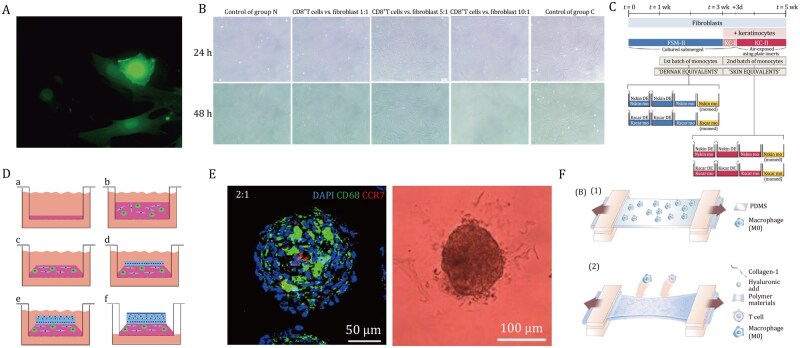
**Applications of immune cell-integrated *in vitro* models in the study of aberrant wound healing**. (A) A hypertrophic scar model established through direct co-culture of human fibroblasts with rat mast cells (illustrating a mast cell exhibiting strong green fluorescence transferring dye into a fibroblast). Source: Reproduced with permission from ref. Foley and Ehrlich (2013) Copyright 2013, Wolters Kluwer Health, Inc. (B) A keloid model generated via indirect co-culture of CD8^+^ T cells and fibroblasts using a transwell system, measured by microscopy at 100×. Source: Reproduced with permission from ref. Shan et al. (2022) Copyright 2022, MDPI. (C) A schematic diagram illustrating the workflow for constructing a three-dimensional keloid model using the artificial dermis MatriDerm^®^ seeded with human peripheral blood mononuclear cells. Source: Reproduced with permission from ref. Limandjaja et al. (2019) Copyright 2019, Springer Nature. (D) A three-dimensional *in vitro* model of diabetic ulcers based on a collagen matrix incorporating monocytes within a transwell culture system. Source: Reproduced with permission from ref. Smith et al. (2021) Copyright 2021, Mary Ann Liebert, Inc. (E) A hypertrophic scar spheroid model containing macrophages, developed using a non-adhesive agarose hydrogel mold. Source: Reproduced with permission from ref. Tan et al. (2020) Copyright 2020, Royal Society of Chemistry. (F) A three-dimensional biomechanical simulation model of keloids incorporating both macrophages and T cells. Source: Reproduced with permission from ref. Zhou et al. (2022) Copyright 2021, John Wiley and Sons.

With the continuous advancement of *in vitro* modeling strategies, organoids have increasingly attracted attention. Based on the concept of spherical organoids, a keloid spheroid model has been developed: patient-derived keloid tissues were first cultured in medium supplemented with bovine serum, buffering agents, and antibiotics, then dissected into approximately 2 mm fragments. Homogeneous spheroids were subsequently selected and seeded into non-adhesive culture plates for further expansion. The biomimetic properties of these spheroids were validated through hallmark features, including type I collagen secretion and TGF-β activation (Dirand et al., 2023). Choi et al. (2024) further co-cultured keloid fibroblasts with endothelial cells at a 4:1 ratio to generate spheroids that mimic a vascularized microenvironment, demonstrating that the incorporation of endothelial cells significantly enhanced the expression of scar-related genes. In the context of wound healing, Virador et al. (2022) cultured murine embryonic fibroblasts in scaffold-free, low-adhesion 96-well plates, where the cells spontaneously formed spheroids. After the administration of test compounds, cell viability was assessed using the 3-(4,5-dimethylthiazol-2-yl)-2,5-diphenyltetrazolium bromide (MTT) assay to evaluate the effects of pharmacological agents on spheroid growth and stability, thereby providing insights into the impact of drugs on wound repair. However, these models largely lacked immune cell components. To address this gap, Tan et al. (2020) innovatively incorporated immune cells into Spheroids (Fig. 5). By co-culturing human macrophages with fibroblasts at varying ratios (ranging from 2:1 to 64:1) in agarose molds, they generated spheroidal constructs that elucidated the mechanism by which M1 macrophages drive fibroblast-to-myofibroblast differentiation. This approach provided a novel platform for studying fibrotic mechanisms and facilitating drug discovery.

Nevertheless, spheroid and organoid-based models also exhibit notable limitations. Their structural architecture restricts nutrient diffusion and cell–cell interactions, with spheroids exceeding 300 μm often developing hypoxia-induced apoptosis. Static culture conditions further lack critical biomechanical forces and dynamic inflammatory gradients. Moreover, inter-patient heterogeneity in gene expression within primary tissues may lead to inconsistent phenotypes, thereby compromising reproducibility and generalizability (Dirand et al., 2023).

### Organ-on-a-chip

Organ-on-a-chip technology combines microfluidic perfusion with engineered tissue constructs, enabling the reconstruction of dynamic skin microenvironments *in vitro* and providing a novel platform for wound-healing research. Early wound-healing skin-on-a-chip investigated the influence of fluid shear stress on cellular behavior but lacked the inclusion of immune components. For instance, Gupta et al. (2022) designed two types of microfluidic chips to explore the role of shear stress in wound repair: one incorporated a silk fibroin membrane embedded at the base to mimic the three-dimensional ECM, where murine fibroblasts were seeded and a “wound” was created by localized trypsin injection, followed by perfusion at different flow rates to generate controllable shear stress, however, this enzyme-based strategy is highly sensitive to flow stability and diffusion effects, resulting in variability in wound geometry and limited reproducibility; the other employed a three-layer structure in which a polydimethylsiloxane (PDMS) barrier was used to create a cell-free gap on the top layer, while fibroblast-seeded silk membranes were placed in the basal channel. Removal of the barrier enabled directional cell migration into the gap, improving geometric control of the wound area, yet introducing additional engineering challenges related to multilayer alignment, sealing reliability, and long-term perfusion stability. By integrating computational fluid dynamics simulations with experimental validation, the authors demonstrated that shear stress critically modulates fibroblast migration and wound closure, with an optimal flow-rate window beyond which excessive shear stress led to cell detachment rather than accelerated wound healing. However, such models included only fibroblasts and lacked the epidermal barrier, endothelial cells, and immune components, while their fabrication required laser micromachining and PDMS molding, which posed a relatively high technical threshold. Given the critical role of immune cells in wound healing, incorporating immune cells into wound-healing chips is essential for recapitulating the sequential inflammatory, proliferative, and remodeling phases, as well as the associated multicellular interactions.

To address this challenge, Biglari et al. (2019) developed a wound-healing chip based on a “vascular-dermal-immune” pattern. This system features a central channel containing a 3D human umbilical vein endothelial cell (HUVEC)-Matrigel microvessel, flanked by side channels populated with fibroblasts and macrophages. The design prevents direct cellular mixing yet preserves essential functional crosstalk among vascular, stromal, and immune components. To overcome the engineering limitation whereby heterogeneous cell distribution within ECM compromises experimental stability and reproducibility, the authors further optimized the original chip architecture by introducing additional inlets for cell and ECM injection and by implementing multi-port access to the lateral channels. Under continuous perfusion, macrophages migrated toward the vascular channel and regulated angiogenesis via the secretion of IL-6 and interleukin-8 (IL-8), thereby recapitulating an early inflammatory multicellular network at the microchip scale. Notably, the model demonstrated that differential M1/M2 polarization distinctly affects both inflammatory intensity and neovascularization, offering a functional platform for screening anti-inflammatory and pro-angiogenic drugs. However, the system did not incorporate keratinocyte-mediated barrier formation. Macrophages were mainly derived from monocyte differentiation rather than tissue-resident sources, and experimental timelines were restricted to no more than 48 hours, thereby excluding the proliferative and remodeling stages. In addition, the ECM composition employed in this platform was fixed and potentially subject to batch-to-batch variability, thereby limiting its capacity to recapitulate dynamic matrix remodeling across different stages of wound healing. Furthermore, the absence of mechanical cues such as tissue tension, cyclic stretch, and physiologically relevant fluid shear stress underscores ongoing engineering challenges in the development of skin wound healing organ-on-a-chip systems. Overall, immune-integrated wound-healing chips remain limited, and systematic studies focusing on chronic wounds or pathological scarring are still lacking (Lebeko et al., 2019).

Notably, immune-integrated skin chips have been applied in the study of various dermatological studies. Representative examples include a full-thickness skin-on-a-chip model incorporating the human promyelocytic leukemia cell line HL-60 neutrophil-like cells and microvascular structure (Kwak et al., 2020); an atopic dermatitis chip integrating the human monocytic cell line U937 to simulate DCs-mediated immune responses (Ramadan and Ting, 2016); and a psoriasis chip embedding human T lymphocytes to investigate transendothelial and transepithelial lymphocyte migration under inflammatory conditions, as well as drug testing (Ren et al., 2021). Such strategies are highly transferrable to wound-healing models. The concept of modular components is equally instructive for the construction of wound-healing models. Building on this notion, Michielon et al. (2024) developed a reusable microfluidic multi-modular adapter (MMA) compatible with standard six-well plates, which enabled the establishment of a three-chamber perfusion system comprising an immune cell reservoir, a full-thickness skin model, and a collection chamber. Collectively, these modules offer a referable engineering toolbox for upgrading wound-healing chips with immunocompetence.

Looking toward the next generation of *in vitro* models, the integration of 3D bioprinting with organ-on-a-chip technology provides a refined approach to reconstructing a stratified architecture. In particular, advanced 3D bioprinting techniques enable the precise fabrication of complex geometries, including perfusable vascular networks, epithelial and endothelial barrier structures, and tissue-specific ECM microenvironments, thereby providing essential structural and physical foundations for recapitulating immune-relevant compartments within organ-on-a-chip systems (Homan et al., 2016; Kim et al., 2013). Such engineered architectures are critical for enabling physiologically relevant simulation of immune cell adhesion, migration, extravasation, and cell-matrix interactions. Maggiotto et al. utilized GelMA hydrogels at varying concentrations in conjunction with sacrificial bioprinting techniques to construct vascular channels within microfluidic platforms (Maggiotto et al., 2025). Although immune cells have not yet been integrated into this system, the platform provides a structural framework for orchestrated immune cell recruitment and resolution over time. Notably, despite the advantages of bioprinting for constructing immune-relevant tissue architectures, the direct bioprinting of immune cells remains a significant engineering challenge. Immune cells are highly sensitive to mechanical stress, shear forces, and processing conditions associated with current printing technologies, which can induce nonspecific activation, alter phenotypic states, or compromise functional viability.

Currently, most skin-on-a-chip models still predominantly rely on cytokine-based biochemical assays or fluorescence labeling strategies to quantify immune responses and track immune cells (Biglari et al., 2019; Kwak et al., 2020). However, these approaches typically require exogenous labeling or are limited to endpoint measurements of soluble factors, thereby precluding genuine real-time and dynamic observation of immune cell states and potentially perturbing cellular function and phenotype. Moreover, labeling procedures may perturb cellular function and phenotype, imposing limitations in multicellular co-culture systems, long-term experiments, and fine-scale dissection of immune cell subpopulations. These constraints underscore an urgent need for label-free, noninvasive monitoring strategies, more advanced integrated sensing technologies, and improved methodologies for immune response quantification. Pérez-Rodríguez et al. introduced a label-free tracking approach that combines real-time phase-contrast microscopy with single-cell trajectory analysis to quantify macrophage migration within collagen matrices in organ-on-a-chip systems, thereby revealing chemotactic dynamics and a concentration-dependent threshold effect in response to immune stimulation. While this label-free strategy provides a generalizable framework for assessing immune cell migration, the authors also identified several limitations, including restricted axial resolution, loss of three-dimensional information due to single-plane imaging, and limited statistical power in multi-condition analyses (Pérez-Rodríguez et al., 2022). The integration of sensing technologies with label-free cell tracking has emerged as a complementary approach for the quantitative assessment of immune function in organ-on-a-chip systems. Ehlers et al. developed the OrganoTEER platform, enabling real-time, continuous monitoring of permeability changes in three-dimensional vascular endothelial barriers via transendothelial electrical resistance (TEER). This system captures inflammation-induced barrier dysfunction without fluorescent probes and supports long-term immune monitoring in immuno-integrated organ-on-a-chip models (Ehlers et al., 2023). Collectively, these approaches provide an essential functional complement to organ-on-a-chip platforms, and their technical frameworks are readily transferable to skin wound models, where they are expected to enhance immune behavior interrogation and model engineering maturity of immune-integrated wound healing organ-on-a-chip systems.

**Figure 6.**
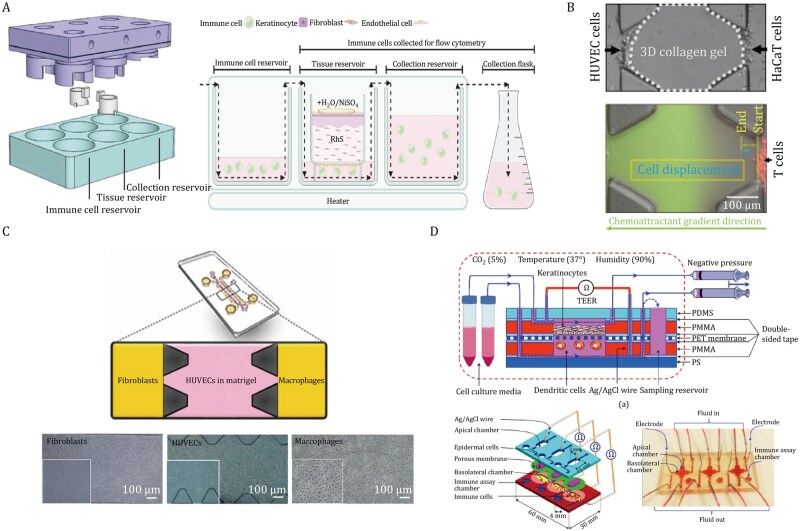
**Applications of immune cell-integrated microfluidic chips in the study of skin diseases and wound healing**. (A) A reusable plug-in skin-on-a-chip device integrated with immune cells. Source: Reproduced with permission from ref. Michielon et al. (2024) Copyright 2024, John Wiley and Sons. (B) A psoriasis organ-on-a-chip model incorporating human T lymphocytes, designed to mimic lymphocyte transmigration across endothelial and epithelial barriers in the inflammatory skin microenvironment and to evaluate novel therapeutic candidates. Source: Reproduced with permission from ref. Ren et al. (2021) Copyright 2022, Royal Society of Chemistry. (C) A wound-healing organ-on-a-chip model integrating macrophages and human umbilical vein endothelial cells. Source: Reproduced with permission from ref. Biglari et al. (2019) Copyright 2018, John Wiley and Sons. (D) An allergic dermatitis organ-on-a-chip model incorporating the human monocytic cell line U937, used to simulate dendritic cell function. Source: Reproduced with permission from ref. Ramadan and Ting (2016) Copyright 2016, Royal Society of Chemistry.

Overall, organ-on-a-chip technology provides a dynamic, quantifiable, and clinically relevant platform for developing *in vitro* wound-healing models with integrated immune functionality (Fig. 6). However, several limitations persist, such as the simplified cellular composition due to the absence of skin appendages and neural elements, donor-to-donor variability that affects reproducibility, and a short culture lifespan—typically less than two weeks—which is inadequate for modeling the full progression of chronic inflammation (Biglari et al., 2019; Gupta et al., 2022; Maggiotto et al., 2025; Michielon et al., 2024). Future advancements will rely on modular designs informed by immune cell dynamics, enabling precise temporal control over the recruitment and resolution of neutrophils, macrophages, and lymphocytes. Structural design should be based on a framework incorporating both epithelial barrier and vascular components. The clinical applicability of such models can be further improved by incorporating patient-derived cells or tissues. From an engineering standpoint, progress is anticipated through the use of three-dimensional bioprinting to fabricate extracellular matrices with tunable stiffness gradients and aligned fiber architecture, the implementation of physiologically relevant shear forces to support long-term perfusion and culture stability, and the development of standardized protocols for comprehensive model characterization. In parallel, emerging strategies for immune cell tracking and functional quantification in organ-on-a-chip platforms, including long-term live-cell imaging, label-free single-cell trajectory analysis, multimodal sensing technologies, and multiscale integrative assessment frameworks, have markedly improved the real-time monitoring and objectivity of immune response evaluation. These methodologies are readily transferable to skin wound healing related disease models and are poised to play a pivotal role in advancing both the mechanistic understanding of immune-driven wound processes and the engineering maturity of immune-integrated skin wound platforms. Nevertheless, important challenges remain, as imaging-induced perturbations, intrinsic limitations in spatial resolution, and the growing complexity of data acquisition and analytical pipelines continue to constrain further improvements in precision and long-term applicability.

## Integration strategies for immune cells in next-generation *in vitro* models

### Strategies for constructing *in vitro* models of aberrant wound healing incorporating immune cells

**Figure 7.**
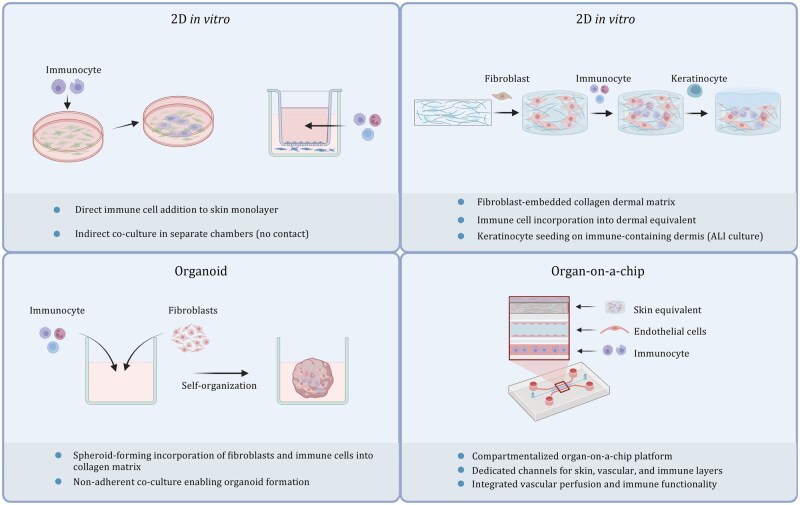
**Strategies for immune-cell integration across *in vitro* skin and wound-healing models**. This figure compares 2D co-culture, 3D static skin equivalents, spheroid/organoid systems, and organ-on-a-chip platforms with respect to immune-cell spatial organization, timing and mode of introduction, cellular composition, and dominant interaction dynamics, including contact-dependent, paracrine, and migration-driven processes. Image was created with BioRender.com, with permission. Abbreviations: 2D, two-dimensional; 3D, three-dimensional; ALI, air-liquid interface.

The primary challenge in developing *in vitro* models that include immune cells is the accurate recapitulation of their origin, timing of infiltration, spatial organization, and interaction dynamics within pathological wound environments (Fig. 7). These models must not only mirror the dynamic processes of wound healing but also reflect the immune features associated with chronic inflammation, fibrosis, and pathological scar formation (Cioce et al., 2024). At the level of cell composition and ratios, various studies have employed different strategies for incorporating immune cells. For example, macrophages and fibroblasts have been co-cultured at defined ratios (ranging from 2:1 to 64:1) to generate 3D spheroidal organoids that mimic a fibrotic microenvironment (Tan et al., 2020). In another experimental setup, fibroblasts and macrophages were co-cultured at a 1:1 ratio in a transwell system, enabling indirect interactions that identified macrophage-driven pathways regulating the fibrotic phenotype of fibroblasts (Kurachi et al., 2021). In models of pathological scar formation, T cells or monocytes have been introduced to fibroblasts at varying ratios (e.g., 1:1 to 10:1), or co-cultured with keratinocytes at a 5:2 ratio, under both direct and indirect contact conditions, thereby demonstrating that fibrotic outcomes are dependent on immune cell interactions (Chen et al., 2019, 2025; Shan et al., 2022; Yang et al., 2024). In terms of temporal and spatial dimensions, numerous models have utilized dynamic strategies for the introduction of immune cells. For instance, in 3D skin equivalent, where immune cells exhibit limited viability *in vitro*, monocytes were added in sequential batches at various stages following dermal layer formation. This strategy not only maintained immune cell infiltration over time but also recapitulated the temporal dynamics of immune cell abundance across distinct phases of wound healing (Limandjaja et al., 2019). In an organ-on-a-chip system, macrophages have been seeded into lateral channels adjacent to fibroblasts, allowing their migration and paracrine signaling to influence the central vascular compartment. These spatially controlled platforms enable the dynamic simulation of immune cell-mediated angiogenesis and inflammatory responses, thereby more accurately mimicking *in vivo* chemotactic and signaling processes (Biglari et al., 2019).

Collectively, these methodological approaches highlight a trend: the integration of immune cells necessitates precise regulation across quantitative, temporal, and spatial parameters to ensure both the pathological relevance and reproducibility of *in vitro* models. Beyond wound models, microfluidic chip technologies have already been utilized in the study of inflammatory skin diseases. For instance, skin-on-chip systems incorporating Langerhans cells have been effectively employed to examine allergen-induced sensitization responses (Ouwehand et al., 2011). These approaches can be adapted for chronic wound research, facilitating the evaluation of immune cell dynamics under diverse pathological conditions. Such cross-disease applications not only contribute to the understanding of common immune mechanisms but also support the development of pathological wound-healing model designs. Nonetheless, substantial challenges persist. Immune cells frequently undergo apoptosis or exhibit phenotypic instability during prolonged *in vitro* culture; static culture systems, which lack vascularization and physiological mechanical forces, are unable to replicate the *in vivo* conditions of tissue tension and perfusion; xenogeneic co-cultures may lead to non-physiological immune interactions; furthermore, although transwell systems are valuable for investigating paracrine signaling, they inherently underestimate the significance of direct cell–cell contact (Chen et al., 2025; Foley and Ehrlich, 2013; Limandjaja et al., 2019). Consequently, future strategies should prioritize advancements in maintaining long-term immune cell viability, refining spatial organization, and enabling systematic comparisons across disease models in order to develop clinically relevant *in vitro* systems for studying aberrant wound healing.

### Model evaluation and validation methods

A high-quality model of pathological wound healing should not only recapitulate the native immune microenvironment during its development but also be subjected to systematic, multi-level evaluation to ensure scientific rigor, reproducibility, and translational relevance. At the histological level, conventional techniques such as hematoxylin and eosin (H&E), Masson, and Sirius Red staining are commonly employed to evaluate dermal thickness, collagen deposition, and fiber alignment. These methods are frequently supplemented with immunofluorescence staining for specific cellular markers, as well as measurements of elastic modulus and graft contraction, to provide a comprehensive assessment of the model’s structural integrity and biomechanical properties (Ibrahim et al., 2014; Smith et al., 2021; Zhu et al., 2016). Some advanced techniques, such as multiphoton fluorescence microscopy, multiplex immunofluorescence combined with tissue-clearing methods, and three-dimensional image reconstruction (Wang et al., 2025; Xu et al., 2024), hold significant potential for high-visualization of collagen networks and precise spatial mapping of immune cell distribution, thereby expanding both the depth and novelty of histological characterization. Functional assays, including transwell migration and scratch wound assays, directly evaluate cellular motility and reparative capacity within the model. Microfluidic organ-on-a-chip platforms offer unique advantages for the dynamic evaluation of immune cell functions, particularly when integrated with sensing technologies that enable real-time and continuous functional readouts. Among these, TEER sensing represents a well-established example of quantitative monitoring of barrier integrity and immune-mediated endothelial responses. Moreover, the platform can be further integrated with microenvironmental sensors, such as dissolved oxygen and pH sensors, as well as *in situ* detection modules for secreted inflammatory mediators, thereby enabling continuous and real-time monitoring and functional validation of the immune microenvironment and inflammatory responses. Such engineering-level integration substantially expands the depth and resolution of model evaluation, allowing immune responses to be assessed in a spatiotemporally resolved, functional, and non-invasive manner (Cognetti and Miller, 2025; Henry et al., 2017; Zhang et al., 2017). At the molecular and signaling level, quantitative real-time polymerase chain reaction (qRT-PCR) and Western blot analyses are routinely employed to detect fibrosis-related genes, inflammatory mediators, and key signaling proteins. Enzyme-linked immunosorbent assay (ELISA), multiplex cytokine assays, and antibody array-based profiling enable comprehensive characterization of cytokine secretion patterns as well as immune cell phenotypic states within immune-integrated wound-healing models. In parallel, matrix remodeling dynamics can be assessed through gelatin zymography (Biglari et al., 2019; Dirand et al., 2024; Kim et al., 2019). Moreover, single-cell RNA sequencing and spatial transcriptomics provide high-resolution profiling of immune cell heterogeneity, spatial distribution, and intercellular crosstalk during aberrant wound repair, offering a sophisticated approach to dissect immune dynamics in engineered models (Thrane et al., 2023). Additionally, metabolomics and proteomics provide valuable insights into the metabolic interactions between immune cells and the ECM, further enhancing the physiological relevance and validation of the constructed model (Nakajima et al., 2024). In this context, Shrestha et al. employed liquid chromatography-tandem mass spectrometry to characterize the macrophage secretome, enabling the simultaneous identification and quantification of thousands of immune-related proteins. Combined with pathway enrichment analysis, this approach elucidated material-induced immunomodulatory networks at a systems level. Such high-throughput molecular profiling strategies provide an important reference for comprehensive monitoring of immune dynamics in organ-on-a-chip platforms (Shrestha et al., 2020).

Beyond traditional evaluation strategies that rely on a single or limited number of evaluation and validation methods, emerging paradigms increasingly emphasize integrated multiscale and multiparametric quantitative analysis. Multiscale analysis enables coordinated interrogation across hierarchical levels from macroscopic tissue architecture to cellular and subcellular organization, while multiparametric strategies integrate structural, functional, molecular, and related dimensions to systematically assess model integrity, functional competence, and biological relevance. Although such unified validation frameworks have not yet been explicitly established in wound healing-specific models, they have been extensively explored in other advanced *in vitro* systems, including organoids and organ-on-a-chip platforms, providing valuable methodological precedents. For instance, Schaart et al. developed a microfluidic platform compatible with high-pressure freezing and correlative light and electron microscopy, enabling long-term live fluorescence imaging of three-dimensional tissues together with ultrastructural analysis at subcellular resolution. Engineering optimization of chip dimensions, materials, and tissue fixation strategies substantially expanded the capacity for interrogation of immune cell-microenvironment interactions within complex tissue construct (Schaart et al., 2025). In another study, Pavesi et al. embedded tumor cells within a collagen matrix in an organ-on-a-chip and introduced human engineered T cells through adjacent channels. By combining real-time three-dimensional imaging and reconstruction, they simultaneously quantified immune cell migration density, invasion depth, and the temporal kinetics of target cell death, in conjunction with multiplex cytokine profiling, allowing functional and molecular characterization of inflammatory signaling networks arising from immune-tissue interactions (Pavesi et al., 2017). In addition, Harter et al. integrated label-free live-cell tracking, multiplex cytokine profiling, and multiplex immunofluorescence imaging together with AI-driven image analysis to achieve systematic quantification of immune responses across temporal, spatial, and cellular-subpopulation dimensions. By partitioning organoids into concentric spatial regions, immune cell distribution and infiltration dynamics were quantitatively mapped, directly linking T cell activation and migration with epithelial cell apoptosis. Collectively, this multiscale, multiparametric quantitative strategy provides a highly reproducible and objective framework for evaluating complex immune responses in organoid-based models (Harter et al., 2024).

**Figure 8.**
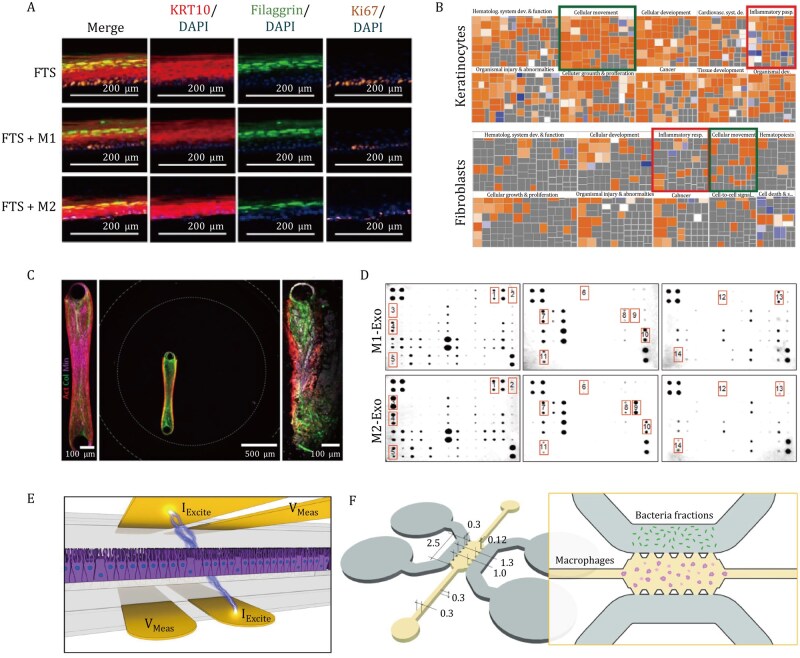
**Multiscale validation strategies for assessing structural integrity, barrier function, and immune activity in advanced skin and wound-healing models**. (A) Multicolor immunofluorescence staining enables spatially resolved assessment of extracellular matrix deposition and immune cell localization within engineered wound-healing models. Source: Reproduced with permission from ref. Lim et al. (2024) Copyright 2024, Institute of Physics Publishing. (B) Transcriptomic profiling combined with Ingenuity Pathway Analysis allows identification of dysregulated signaling pathways associated with aberrant wound repair. Source: Reproduced with permission from ref. Kühbacher et al. (2017) Copyright 2017, Oxford University Press. (C) Correlative light and volume electron microscopy enables three-dimensional, high-resolution characterization of tissue architecture and matrix organization, providing a powerful tool to examine immune cell spatial distribution within immune-integrated wound-healing models. Source: Reproduced with permission from ref. Schaart et al. (2025) Copyright 2025, Springer Nature. (D) Antibody array-based profiling enables multiplexed characterization of immune cell phenotypic states within immune-integrated wound-healing models. Source: Reproduced with permission from ref. Kim et al. (2019) Copyright 2019, John Wiley and Sons. (E) Transepithelial electrical resistance measurement reflecting quantitative, real-time assessment of tight junction integrity and barrier function under dynamic culture conditions. Source: Reproduced with permission from ref. Henry et al. (2017) Copyright 2018, Royal Society of Chemistry. (F) Quantitative assessment of immune cell migration dynamics, directional responsiveness, and phenotype-specific behavior within three-dimensional microfluidic environments provides robust validation of immune cell activity and functional fidelity in engineered tissue models. Source: Reproduced with permission from ref. Pérez-Rodríguez et al. (2022) Copyright 2022, Springer Nature.

In summary, only through an integrated evaluation framework—shifting from single or limited endpoints toward multiscale and multiparametric quantitative assessment, encompassing histological, cellular, molecular, and functional assessments—can the fidelity and robustness of pathological wound healing models be guaranteed. Such a framework enables coordinated interrogation of tissue architecture, immune dynamics, and molecular signaling across hierarchical spatial and temporal scales, thereby establishing a solid foundation for mechanistic studies and preclinical drug discovery. Looking ahead, the development of AI and machine learning-based multimodal data integration platforms (Kalasin et al., 2022) may facilitate systematic analysis and predictive modeling across diverse parameters, ultimately improving the translational utility of wound healing models in biomedical research (Fig. 8).

## Advantages, challenges, and future perspectives

Incorporating immune cells into *in vitro* models of impaired wound healing enhances structural and functional fidelity, more closely recapitulating the pathological microenvironment of the diseases. This approach offers distinct advantages for mechanistic investigations and drug discovery (Bergers et al., 2016). First, these models enable the reconstruction of a skin barrier while simultaneously integrating immune response networks, allowing researchers to dissect the dynamic interactions between immune cells and structural cells under controlled conditions. For example, the incorporation of M1/M2 macrophages, T cell subsets, mast cells, or Langerhans cells into three-dimensional skin equivalents or skin-on-chip platforms can recapitulate specific immune states associated with the inflammatory, reparative, or fibrotic phases (Morsink et al., 2020). Second, such models permit investigation across multiple scales—from tissue-level outcomes (barrier function, collagen deposition, tissue thickness) to cellular behaviors (immune cell infiltration, activation status, migratory patterns), and down to molecular pathways (signal transduction, cytokine profiling)—thereby providing a reproducible experimental platform for validating immune response mechanisms.

Despite these advances, immune cell-integrated *in vitro* models still face significant challenges in their construction and application. Although the addition of immune cells to *in vitro* systems is relatively straightforward, the central challenge lies in enabling immune cells to function in a physiologically relevant manner without compromising the structural integrity, experimental controllability, and analytical tractability of the original model—an issue that represents both a biological and an engineering challenge. From a biological perspective, in most current models, immune cells and structural cells, such as keratinocytes or fibroblasts, are sourced from unmatched donors, a situation that may elicit allogeneic immune responses and unintended inflammatory reactions. In particular, when adaptive immune cells are incorporated, human leukocyte antigen (HLA) mismatch may induce non-specific immune activation resembling graft-versus-host disease (GVHD)-like responses, thereby constituting a major limitation related to cell sourcing (Morrison et al., 2024). Furthermore, constructing 3D models that incorporate immune cells requires multiple cell types, involves prolonged and technically complex culture procedures, and remains highly dependent on operator expertise. Donor-dependent factors, including donor age and health status, together with inter-donor heterogeneity, introduce substantial variability in immune response magnitude, leading to batch-to-batch inconsistency and compromised reproducibility (Koning et al., 2021). Additionally, during prolonged *in vitro* culture, immune cells are susceptible to phenotypic drift or apoptosis, which limits their capacity to maintain *in vivo*-like functionality and thereby constrains long-term observations or modeling of chronic wound processes (Bisconti et al., 2025).

From an engineering perspective, faithfully recapitulating the dynamic behavior and functional maintenance of immune cells requires the design of highly biomimetic microenvironments. Despite substantial advances in bioprinting, organoid technologies, and organ-on-a-chip platforms, the development of immune-integrated *in vitro* models remains constrained by limitations in material selection, model architecture, and mechanical regulation. The ECM composition and device substrate materials critically influence immune cell behavior during model construction, an effect that is particularly pronounced in organ-on-a-chip systems. The ECM, beyond serving as a structural scaffold, actively regulates immune cell migration, activation, and phenotype through its mechanical properties and biochemical cues. For instance, neutrophils embedded in basement membrane extract-based Geltrex matrices exhibit spontaneous migratory behavior even in the absence of externally imposed chemotactic gradients, whereas migration within pure type I collagen matrices requires additional chemotactic factors to be initiated (Riddle et al., 2022). Currently, the most widely used ECM materials include Matrigel, fibrillar type I collagen, gelatin-derived hydrogels, natural polysaccharide-based matrices, among others. While these materials facilitate three-dimensional tissue construction, each presents inherent limitations. Basement membrane-derived matrices such as Matrigel suffer from undefined composition, significant batch-to-batch variability, and limited long-term stability, introducing uncontrollable variables that compromise reproducibility and quantitative reliability of the immune integrated models (Bu et al., 2022); Fibrillar ECMs such as type I collagen offer greater compositional stability but provide limited tunability in mechanical properties and biological variability. Gelatin-based hydrogels (e.g., GelMA) and natural polysaccharides are more suitable for long-term wound-healing models, yet may deviate from physiological ECM characteristics, potentially affecting immune relevance (Skardal, 2024). In addition to ECM selection, device materials exert a profound influence on immune cell function. The PDMS, which is widely used in organ-on-a-chip fabrication, is known to nonspecifically absorb hydrophobic small molecules, including certain drugs and signaling mediators, thereby perturbing pharmacokinetic readouts and destabilizing cytokine gradients. Moreover, uncrosslinked oligomers may leach from PDMS, inducing chronic cytotoxic effects, while long-term culture can lead to structural deformation and gas permeability issues that compromise environmental control and reduce the stability and reproducibility of immune responses (Shrimali et al., 2025; Toepke and Beebe, 2006). Beyond PDMS, further studies have demonstrated that distinct biomaterial surfaces, including degradable polar hydrophobic ionic polymers, poly (lactic-co-glycolic acid) (PLGA), and tissue culture polystyrene, can alter macrophage secretory profiles and bias cells toward pro-inflammatory or pro-regenerative phenotypes. These findings underscore that material constitutes a form of implicit immunomodulation in immune-integrated organ-on-a-chip systems; without systematic engineering evaluation, such effects may lead to biased or misleading interpretations of immune behavior (Shrestha et al., 2020). From a model-design standpoint, simply incorporating immune cells does not equate to reconstructing physiologically relevant immune-repair processes. Wound healing involves coordinated immune cell recruitment, transendothelial migration, movement within the ECM, and dynamic interactions with epithelial and stromal cells—processes that are highly sensitive to spatial organization and engineering design (Van Os et al., 2023). Conventional two-dimensional cultures or simplified three-dimensional co-culture systems typically lack functional barriers, gradient control, and authentic migration pathways, resulting in immune cell behavior that is dominated by nonphysiological, passive interactions arising from random motion or gravity-induced sedimentation, rather than spatially guided migration. In contrast, microfluidic platforms, through the integrated coupling of perfusion channels, endothelial barriers, and three-dimensional ECMs, can support key steps of immune trafficking, including rolling, adhesion, transendothelial migration, and directional interstitial infiltration, thereby more closely approximating *in vivo* wound healing processes. Nevertheless, many available organ-on-a-chip platforms rely on membrane-based compartmentalization, pillar-array structures, or fixed barrier-separated parallel microchannels to achieve tissue separation. While these inert structural elements facilitate fluidic control and manufacturing robustness, they can inadvertently obstruct direct cell–cell interactions and limit the ability of immune cells to undergo authentic infiltration, cross-interface migration, and dynamic tissue interactions within the chip (Olaizola-Rodrigo et al., 2025). Furthermore, wound healing is a highly mechanosensitive process. Although microfluidic models commonly incorporate flow-induced shear stress and mechanical stimulation, precise hydrodynamic control remains a major challenge in immune-integrated contexts. Immune cells are exquisitely sensitive to matrix stiffness, porosity, viscoelasticity, and shear forces; deviations from physiological ranges may result in aberrant activation or adhesion behavior. Many organ-on-a-chip systems struggle to maintain flow stability during long-term perfusion and are susceptible to bubble formation, introducing variability across experiments. In addition, differences in microchannel geometry and surface properties across platforms mean that identical nominal flow rates can generate different shear stresses at the cellular level, undermining cross-platform reproducibility and quantitative comparability of immune responses (Dabbagh Moghaddam et al., 2025; Khanmohammadi et al., 2025).

**Figure 9.**
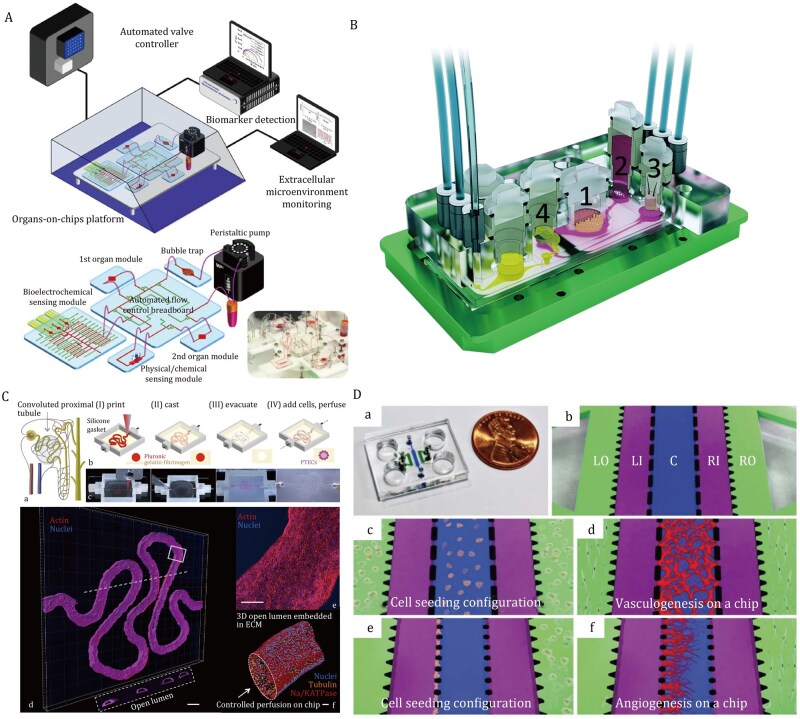
**Foundational engineering innovations across advanced organ-on-a-chip systems informing next-generation skin wound healing models**. (A) A multimodal sensor-integrated organ-on-a-chip platform enables in situ, continual, and automated monitoring of microenvironmental biochemical and biophysical dynamics, and can be leveraged to support dynamic assessment of immune-tissue interactions in immune-integrated wound-healing models. Source: Reproduced with permission from ref. Zhang et al. (2017) Copyright 2017, National Academy of Sciences. (B) A modular four-organ chip achieves physiologically scaled co-culture of intestine, liver, skin, and kidney models for *in vitro* systemic drug testing, and offers a versatile framework for exploring immune-mediated inter-organ crosstalk relevant to wound-healing. Source: Reproduced with permission from ref. Maschmeyer et al. (2015) Copyright 2015, Royal Society of Chemistry. (C) Integration of 3D bioprinting with organ-on-a-chip platforms enables programmable construction of complex tissue architectures with perfusable functionality, providing a transferable strategy to increase spatial resolution and architectural complexity in the design of immune-integrated wound-healing models. Source: Reproduced with permission from ref. Homan et al. (2016) Copyright 2016, Springer Nature. (D) A microfluidic co-culture platform enables self-assembled, perfusable 3D microvascular networks with stable barrier function and physiological shear responsiveness, and is well suited for modeling immune cell trafficking and vascular-immune interactions in wound-healing contexts. Source: Reproduced with permission from ref. Kim et al. (2013) Copyright 2013, Royal Society of Chemistry.

Looking ahead, immune cell-integrated wound healing models are expected to enable substantive advances across multiple dimensions (Fig. 9). At the level of structural design, appropriately engineered microfluidic configurations are essential for recapitulating physiologically relevant immune behaviors. By incorporating endothelialized perfusion channels to mimic vascular barriers and spatially separating tissue compartments from circulating flow domains, while precisely controlling shear stress as well as stable chemical and oxygen gradients, a single platform can support both the functional segregation and coordinated interaction of circulating immune cells and tissue-resident cells. Such designs enable the reproduction of complex immune behaviors observed *in vivo*, including immune cell rolling, adhesion, transendothelial migration, and directed interstitial migration, thereby simulating the dynamic trafficking of immune cells between the vasculature and tissue. Importantly, this dynamic configuration avoids the nonphysiological amplification of immune effector functions commonly observed in static co-culture systems (Rogal et al., 2022). Beyond microfluidic architecture, incorporating the interactions between immune cells and other pathological factors—such as bacterial infection (Shan et al., 2024) or mechanical stress (Jiang et al., 2024)—while also integrating complex structures like nerves, hair follicles, and sebaceous glands into microfluidic chips (Vidal et al., 2019) or skin equivalents may enable a more comprehensive reconstruction of the wound healing immune microenvironment. In parallel, reducing inert physical barriers and reconstructing authentic migration pathways has emerged as a key design direction for next-generation organ-on-a-chip platforms. Using the OrganoPlate microfluidic platform, Riddle et al. developed a membrane-free three-dimensional endothelial-ECM co-culture system in which neutrophils, under continuous perfusion, could sequentially undergo endothelial adhesion, transmigrate across the endothelium, and subsequently migrate within the matrix. In the absence of fixed physical barriers, immune cell migration trajectories more closely resembled those observed during *in vivo* inflammation (Riddle et al., 2022). Such designs enhance the physiological relevance of immune cell motility and are valuable for modeling the inflammatory phase of wound healing, during which immune cell functions are highly active. When combined with 3D printing technologies capable of fabricating complex geometries, including endothelial conduits, compartmentalized regions, and spatially defined extracellular matrices, these platforms further expand design flexibility and precision (de Barros et al., 2025).

To construct more comprehensive “human physiology-substituting” models that incorporate immune function, hybrid platforms have emerged, including organoid-on-a-chip systems and multi-organ-on-a-chip configurations. Organoids offer clear advantages in recapitulating tissue architecture and multicellular lineage diversity, whereas organ-on-a-chip platforms provide superior control over fluid dynamics and microenvironmental parameters. Embedding patient-derived organoids within microfluidic chips enables the integration of tissue specificity with dynamic regulation, a strategy that has already been validated in personalized cancer immunotherapy assessment. Such models have demonstrated the ability to capture pronounced inter-individual differences in immune cell infiltration, effector intensity, and cytokine release, driven by factors including target antigen expression, anatomical origin of the organoid, and donor-specific immune backgrounds (Maulana et al., 2024). These findings highlight the capacity of human-based immune organoid models to capture individual variability that is difficult to reproduce in animal models or conventional 2D cultures, providing powerful tools for immunotherapy evaluation, immune-related drug and biomaterial screening, and personalized precision medicine. Concurrently, immune responses *in vivo* often involve coordinated regulation across multiple organs and systemic feedback mechanisms. Accordingly, multi-organ-on-a-chip systems hold substantial promise for simulating cross-organ immune dynamics. By connecting multiple organ chips in series or parallel within a shared circulation loop, tissues can communicate via secreted factors (Maschmeyer et al., 2015), and in some cases immune cells can migrate between different organ compartments, establishing a more integrated and physiologically relevant system. Sasserath et al. developed a gravity-driven multi-organ-on-a-chip platform incorporating three functional human tissue modules—liver, cardiac muscle, and skeletal muscle—together with an innate immune component, in which the human monocytic cell line THP-1 were continuously maintained within the circulation to model circulating monocyte or macrophage populations. By selectively inducing local or systemic immune activation, this platform revealed tissue-specific immune cell recruitment, distinct polarization states, and differential impacts on organ function (Sasserath et al., 2020). Such multi-organ systems demonstrate immune complexities that are difficult to recapitulate using single-organ models and provide valuable tools for investigating systemic inflammatory responses and potential off-target effects associated with wound healing. Notably, in April 2025, the U.S. Food and Drug Administration (FDA) announced plans to gradually phase out conventional animal testing in favor of drug safety assessment systems based on organoids and organ-on-a-chip technologies. In the same month, the National Institutes of Health (NIH) emphasized the prioritization of human-based research technologies—including organoids, tissue chips, and computational models—to reduce reliance on animal models. These human-derived platforms preserve species-specific immune responses, tissue architecture, and inter-individual variability, features that are critically important in disease modeling and represent intrinsic advantages for model development. For example, a study combining immune cells from multiple donors with intestinal organoids has revealed pronounced donor-dependent differences in immune-mediated epithelial injury, driven by factors such as target antigen expression, organoid anatomical origin, and donor-specific immune backgrounds (Harter et al., 2024). Such finding demonstrates that human-based models can capture inter-individual variability that is difficult to recapitulate in animal models or conventional two-dimensional cultures, providing powerful tools for immunotherapy evaluation, immune-related drug and biomaterial screening, and personalized precision medicine. Against this policy backdrop, immune cell-containing *in vitro* models, combined with advances in 3D bioprinting, organoid engineering, and organ-on-a-chip systems, are poised to become valuable tools in drug and biomaterial safety evaluation, accelerating the clinical translation of wound healing therapeutics. Furthermore, standardized and scalable immune-augmented skin models will provide robust support for high-throughput drug screening and multifactorial mechanistic investigations (Kang et al., 2022). In addition, established strategies from immune-based models of other skin diseases, such as psoriasis and atopic dermatitis (De Vuyst et al., 2017; Ubago-Rodríguez et al., 2024), can be adapted to aberrant wound healing research, offering new opportunities to explore immune cell behaviors across different pathological contexts and identify shared regulatory pathways.

## Conclusion

Aberrant wound healing represents a significant clinical challenge, involving not only cellular proliferation and ECM remodeling but also the recruitment, activation, and functional polarization of immune cells. Traditional *in vitro* models have advanced our understanding of wound repair mechanisms to some extent; however, they largely neglect the reconstruction of the immune microenvironment, thereby limiting their ability to faithfully recapitulate the complex and dynamic processes of tissue repair. Consistent with this, a key takeaway from recent research is that 'adding immune cells' alone is insufficient; rather, disease-relevant modeling requires that immune cells be embedded within physiologically meaningful transport, barrier, and three-dimensional migration contexts. Recent advances in *in-vitro* modeling and bioengineering have enabled the integration of immune cells into skin equivalents and organ-on-a-chip systems, offering more physiologically relevant platforms for studying wound healing. The incorporation of immune cells into models of aberrant wound repair has emerged as a critical innovation in the field.

Multiple strategies have been investigated to integrate immune cells into aberrant wound healing models, including the establishment of immune cell layers or the implementation of direct and indirect co-culture approaches within organoids, skin equivalents, and microfluidic organ-on-a-chip platforms. These models more accurately recapitulate the dynamic infiltration and functional polarization of immune cells, providing valuable insights into the mechanisms underlying inflammation-driven aberrant repair. Crucially, immune-integrated platforms are increasingly shifting from static endpoint measurements toward continuous, quantitative assessment of immune dynamics, covering rolling, adhesion, trans-barrier extravasation, directional interstitial migration, and effector interaction. Nevertheless, current systems still face several limitations, including the short lifespan and phenotypic instability of immune cells, as well as the challenges associated with incorporating multiple skin appendages, which these factors restrict their applicability in long-term studies and high-throughput applications. Beyond these biological constraints, engineering limitations remain decisive bottlenecks for reproducibility and translation, including material- and interface-dependent immune modulation, the high mechanical sensitivity of immune cell behavior to non-physiological flow and force regimes, limitations in sustaining physiologically relevant transport environments, and insufficient standardization across platforms. Furthermore, next-generation immune-integrated wound-healing models will be defined not only by cellular composition, but equally by their capacity to track and characterize immune dynamics. The convergence of label-free sensing, advanced imaging, and computational analysis will enable standardized and longitudinal quantification of immune behavior within complex three-dimensional tissues.

Looking forward, next-generation immune-integrated *in vitro* aberrant wound healing models are expected to achieve substantial advancements. Emerging technologies such as three-dimensional bioprinting, organoid self-assembly, and automated microfluidic culture are anticipated to enhance model complexity and stability, enabling the integration of vascular, neural, and appendage structures to more comprehensively reconstruct the immune microenvironment. Organoid-on-a-chip and multi-organ immune platforms further extend this framework to patient-relevant and system-level immune responses beyond single-tissue models. Concurrently, international policy trends are promoting high-tech research systems, with agencies such as the FDA and NIH advocating for the reduction of animal experimentation and the prioritization of organ-on-a-chip and organoid technologies. These developments create opportunities for human-based immune-integrated wound healing models to be incorporated into drug discovery and safety evaluation pipelines. Balancing human heterogeneity with experimental consistency will be a central design principle, particularly for patient-derived, precision-oriented applications. In conclusion, immune cell-integrated *in vitro* models of aberrant wound repair hold irreplaceable value in basic research and possess significant potential for drug screening, regenerative therapy testing, and clinical translation. With the convergence of multidisciplinary technologies and supportive policy initiatives, these models are poised to provide a robust experimental foundation for the development of precision therapeutics for aberrant wound healing.

## Artificial intelligence (AI)

Artificial intelligence-assisted tools were used for language refinement. All scientific content was critically reviewed and approved by the authors.

## Abbreviations:

TGF-β: transforming growth factor beta
IL-1: interleukin-1
IL-6: interleukin-6
TNF-α: tumor necrosis factor alpha
NETs: neutrophil extracellular traps
Tregs: regulatory T cells
IL-1β: interleukin-1 beta
2D: two-dimensional
3D: three-dimensional
DCs: dendritic cells
VEGF: vascular endothelial growth factor
iNKT: invariant natural killer T cells
IL-10: interleukin-10
iNOS: inducible nitric oxide synthase
ECM: extracellular matrix
Th: T helper
DFUs: diabetic foot ulcers
MTT: 3-(4,5-dimethylthiazol-2-yl)-2,5-diphenyltetrazolium bromide
PDMS: polydimethylsiloxane
HUVEC: human umbilical vein endothelial cell
IL-8: interleukin-8
MMA: multi-modular adapter
TEER: transendothelial electrical resistance
H&E: Hematoxylin and eosin
qRT-PCR: quantitative real-time polymerase chain reaction
ELISA: enzyme-linked immunosorbent assay
HLA: human leukocyte antigen
GVHD: graft-versus-host disease
PLGA: poly(lactic-co-glycolic acid)
FDA: U.S. Food and Drug Administration
NIH: National Institutes of Health
THP-1: human acute monocytic leukemia cell line
ALI: air-liquid interface
